# Real-world evidence of the intrinsic limitations of PCR-based EGFR mutation assay in non-small cell lung cancer

**DOI:** 10.1038/s41598-022-17394-7

**Published:** 2022-08-09

**Authors:** Chia-I Shen, Chi-Lu Chiang, Tsu-Hui Shiao, Yung-Hung Luo, Heng-Sheng Chao, Hsu-Ching Huang, Chao-Hua Chiu

**Affiliations:** 1 Department of Chest Medicine, Taipei Veterans General Hospital, 201, Section 2, Shih-Pai Road, Taipei, 11217 Taiwan; 2 School of Medicine, College of Medicine, National Yang Ming Chiao Tung University, 155, Section 2, Linong Street, Taipei, 112 Taiwan; 3 Institute of Clinical Medicine, College of Medicine, National Yang Ming Chiao Tung University, 155, Section 2, Linong Street, Taipei, 112 Taiwan; 4 Taipei Cancer Center, Taipei Medical University Hospital, 252, Wuxing Street, Taipei, 110 Taiwan

**Keywords:** Cancer genetics, Lung cancer, Next-generation sequencing

## Abstract

Detection of driver gene mutations is important in advanced NSCLC. The cobas EGFR mutation test is a mutant allele-specific real-time PCR assay with limitation owing to its primer design. Next-generation sequencing-based assay has a higher mutation detection coverage; however, its clinical impact remains unclear. We retrospectively collected the records of stage IV NSCLC patients with wild-type EGFR tested by cobas test. FoundationOne CDx was used for comprehensive genomic profiles. We then evaluated the missed EGFR mutations by the cobas test. We studied 62 patients. The median age was 60 (range: 35–86 years). Most patients were male and 58.1% were smokers. 91.9% were adenocarcinomas. Of the 62 samples, 7 (11.3%) were detected with EGFR mutations by NGS. Among these overlooked EGFR mutations, five were exon 20 insertions, and two were exon 19 deletions. Two patients received EGFR TKIs and showed durable response with PFS 5.9 months and 10.1 months, respectively. Using NGS as the standard, the false-negative rate of the cobas EGFR mutation test was 11.3%—in a population with a high prevalence of EGFR mutations. The most overlooked mutations were exon 20 insertions. A comprehensive EGFR mutation assay can provide significant benefits to patients with NSCLC.

## Introduction

The development of immunotherapy and targeted therapies have greatly improved the treatment of advanced non-small cell lung cancer (NSCLC). Novel drugs targeting driver genes, including epidermal growth factor receptor (EGFR), anaplastic lymphoma kinase (ALK), and ROS1, have been developed and widely applied in clinical practice^1–8^. Identifying actionable driver mutations has become a decisive step in cancer diagnosis^9–11^. Among all well-known driver mutations, EGFR mutations are highly prevalent in East Asia and account for over 50% of lung adenocarcinoma cases in Taiwan^12^. The detection of EGFR mutations is the first step in molecular analysis in this area. Earlier, Sanger sequencing was the gold standard; however, it is tedious and shows suboptimal sensitivity^13,14^. The cobas EGFR mutation test is a highly sensitive, mutant allele-specific real-time PCR method that uses pre-designed primers to detect specific mutations. The assay improved the detection rate, reduced turnaround time, and substituted Sanger sequencing as the new standard^15–17^. However, several uncommon mutations lying outside the coverage area of the pre-designed primers may be missed^18^, compromising the patients’ clinical outcomes. Next-generation sequencing (NGS) is a more comprehensive technique with wider mutation coverage. It not only detects EGFR mutations but also provides genetic profiles of the tumors^19,20^. In this study, we used NGS as the standard and determined the false-negative rate and intrinsic limitations of the cobas EGFR test, and investigated its clinical significance.

## Materials and methods

### Patient cohort

We retrospectively collected the medical records of patients with stage IV NSCLC diagnosed at a tertiary medical center in Taiwan. During the study period, the standard molecular test for newly diagnosed, metastatic, non-squamous cell lung carcinoma patients consisted of EGFR sequencing and ALK immunohistochemical staining. Clinically, some EGFR/ALK double-wild-type patients might undergo NGS testing to identify potentially actionable mutations. The study sample of two different sequencing tests may not be the same specimen under clinical practice. Patients who had qualified NGS results were included for further analysis. The data were collected between January 1, 2012, and July 31, 2020. All experiments were performed in accordance with the relevant guidelines and regulations. This study was approved and the informed consent was waived by the Institutional Review Board of Taipei Veterans General Hospital (2021-02-010CC).

### Allele-specific real-time PCR mutation assay

The cobas EGFR mutation test v2 (Roche Molecular Diagnostics, Pleasanton, CA, USA) is a high-sensitivity allele-specific real-time PCR test that detects mutations in EGFR^16^. The assay was designed to detect 42 most common EGFR mutations by using specific oligonucleotide probes. The detection coverage included 29 exon 19 deletions, five exon 20 insertions, three G719X missense mutations, two L858R missense mutations, and S768I, T790M, and L861Q missense mutations. The mutation test included two steps: extract DNA from tissue and PCR amplification. DNA was extracted from formalin-fixed, paraffin-embedded (FFPE) tissue sections. Genomic DNA was then used for testing using a cobas 4800 analyzer. The allele-specific primers target the mutation sequences during amplification^16^. The procedures were performed according to the manufacturer’s standardized protocols at the institute in the molecular laboratory, Department of Pathology.

### Next-generation sequencing

NGS was performed for all patients using FoundationOne CDx (Foundation Medicine, Cambridge, MA, USA) in a commercial laboratory using FFPE tissue sections. The specimens were required for 10 unstained slides cut at 4–5 microns thick and meet the minimum surface area 25 mm^2^. The optimal tumor content was 30% and the minimum was 20%. All specimens were sent for quality control check-up. DNA was extracted, fragmented, and the whole-genome shotgun library was constructed. The specimens were deep-sequenced using Illumina HiSeq 4000 platform. The assay detects deletions, insertions, substitutions, and copy number alterations in 324 genes. Additionally, the test reports genomic signatures, including microsatellite instability and tumor mutation burden.

### Clinical data collection

We evaluated the patients’ clinical characteristics and mutation profiles. The following data were collected: sex, age, smoking history, ECOG performance status at the time of diagnosis, staging, diagnostic tools, date of the pathology report, EGFR mutation status, tumor percentage of the tissue slide, clinical laboratory data, types of systemic and local treatment, and clinical outcomes. We evaluated the false-negative rate of cobas EGFR mutation test using NGS. Patients’ clinical pathways and outcomes were investigated, especially those with false-negative EGFR mutations. We defined driver mutations as those with potential targeted therapies, including EGFR, BRAF(V600E), and KRAS(G12C) missense mutations; ALK, ROS1, RET, and NTRK fusions; and MET exon 14 skipping. The response to treatment was assessed by clinical physicians. Time to treatment failure (TTF) was defined as the period between the initiation of treatment and documented treatment failure. Overall survival (OS) was defined as the date from the initial diagnosis to the last follow-up or death. We used the eighth edition of the American Joint Committee on Cancer staging system for lung cancer staging.

### Statistics

The patients’ baseline characteristics were presented using descriptive statistics. We performed chi-square or Fisher’s exact tests for categorical variables. Continuous variables were compared using Student’s t-test or Mann–Whitney U test. A two-tailed p-value < 0.05 indicated statistical significance. SPSS software (version 21.0, IBM, USA) was used for all analyses.

### Ethical disclosure

This study was approved by the Institutional Review Board of Taipei Veterans General Hospital (2021-02-010CC).

## Results

### Patient characteristics

A total of 87 patients were enrolled during the study period. NGS was not performed for 25 (28.7%) patients from whom adequate tissue samples could not be obtained or extracted genomic DNA did not pass quality control check-ups. Hence, only 62 patients were included for further evaluation. The median age was 60 years (range: 35–86 years). Most patients had an ECOG performance of 0 or 1. Two-thirds of the cohort (66.1%) were male, and over half of the patients (58.1%) were smokers. Lung adenocarcinoma was the predominant histological type (91.9%). Specimens were collected via a needle (62.9%) or surgical (37.1%) biopsy (Table 1). Of all the 62 specimens, 48 specimen (77.4%) were obtained from the same specimen for both cobas EGFR test and NGS study.

**Table 1:** Patient characteristics (n = 62).

Characteristics	Number (%)
Median age (range)	60 (35–86)
**Gender**	
Male	41 (66.1)
Female	21 (33.9)
**Smoking status**	
Non-smoker	26 (41.9)
Smoker	36 (58.1)
**Histology**	
Adenocarcinoma	57 (91.9)
Non-small cell carcinoma, NOS*	5 (8.1)
**ECOG**	
0–1	59 (95.2)
≥2	3 (4.8)
**Sampling method**	
Surgical biopsy	23 (37.1)
Needle biopsy	39 (62.9)

*NOS not otherwise specified.

### NGS study

**Figure 1:**
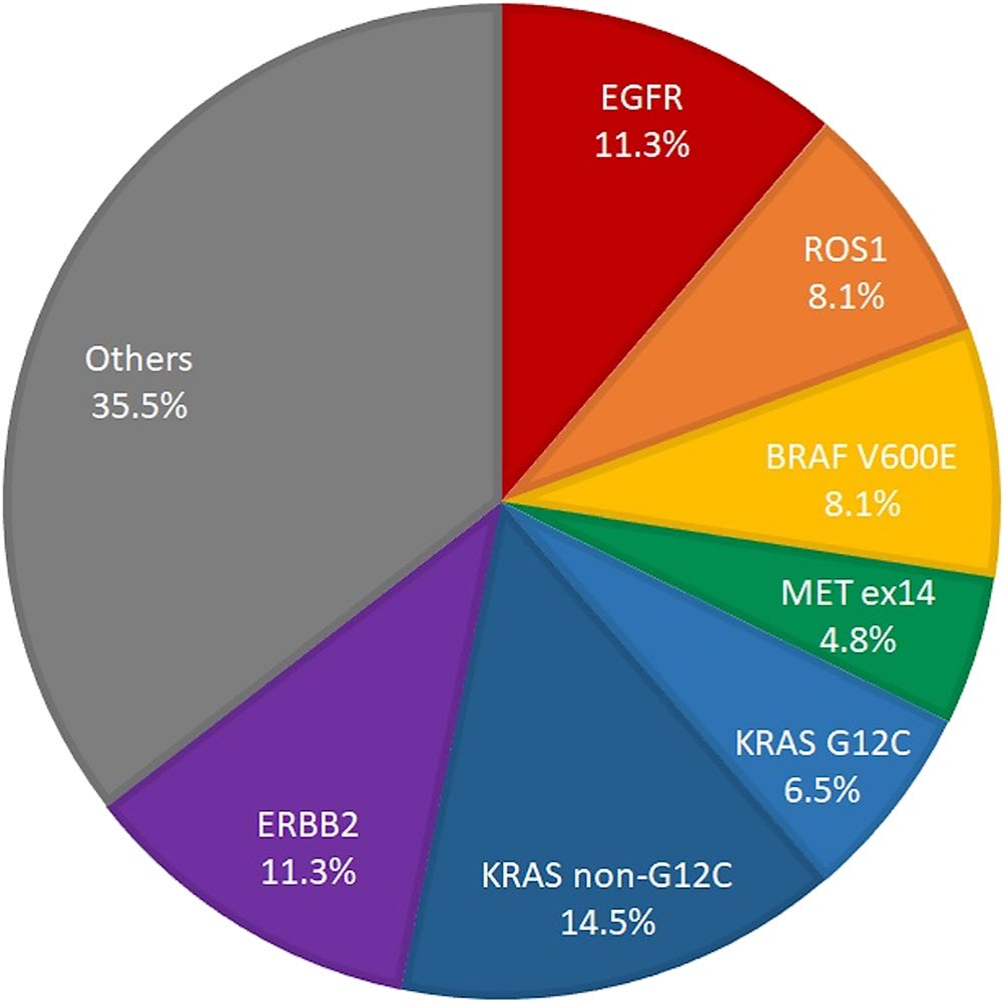
Driver mutations detected by NGS in the cobas EGFR wild type cohort (n = 62).

A total of 24 (38.7%) patients had driver mutations detected by NGS in the EGFR/ALK double wild-type cohort (Fig. 1). EGFR mutations, missed by the cobas EGFR test, were found in seven patients (11.3%). Other potential actionable genetic alterations detected included ROS1 fusions in 5 (8.1%), BRAF V600E mutations in 5 (8.1%), MET exon 14 skipping mutations in 3 (4.8%), KRAS mutations in 13 (21.0%), and ERBB2 mutations in 7 (11.3%) patients. In the KRAS group, G12C was the predominant type (n = 4), accounting for 6.5% of the EGFR/ALK double wild-type cohort. Moreover, ERBB2 mutations were found in 7 (11.3%) patients and gene amplification (such as MET, ERBB2, and FGFR) and other clinically significant mutations (such as TP53, STK11, SMAD4 and SMARCA4) were identified in 22 (35.5%) patients and were categorized into the non-driver mutations group.

### Profiling under-detected EGFR mutations

The seven (11.3%) samples with false-negative cobas EGFR results were all adenocarcinoma. Five were obtained from needle biopsies and two from surgical biopsies. Most were exon 20 insertions (5/7, 71.4%), while the others were exon 19 deletions (2/7, 28.6%). In the exon 20 insertion group, there were two A763_Y764insFQEA, one N771_P772insN, one V769_D770insGTV, and one H773_V774insNPH. The two exon 19 deletions were L747_A750 > P and T751_I759 > S. The details are summarized in Table 2. All these mutations were out of the detection coverage by the cobas EGFR mutation test v2 except L747_A750 > P.

**Table 2:** Profiles of overlooked EGFR mutations.

No	Gender	Smoking	Histology	EGFR mutation status		EGFR-TKI	Line of therapy	Response	TTF (m)	OS (m)
#1	Female	Non-smoker	Adenocarcinoma	A763_Y764insFQEA	ex20ins	Erlotinib	Second-line	SD	5.9	19.9
#2	Male	Non-smoker	Adenocarcinoma	N771_P772insN	ex20ins					
#3	Female	Non-smoker	Adenocarcinoma	V769_D770insGTV	ex20ins					
#4	Female	Non-smoker	Adenocarcinoma	A763_Y764insFQEA	ex20ins					
#5	Female	Non-smoker	Adenocarcinoma	L747_A750 > P	ex19del					
#6	Male	Smoker	Adenocarcinoma	H773_V774insNPH	ex20ins					
#7	Female	Non-smoker	Adenocarcinoma	T751_I759 > S	ex19del	Erlotinib	Second-line	SD	10.1	33.8
						Osimertinib	Fifth-line	SD	10.9	–

### Clinical outcomes

Of the seven patients with false-negative EGFR, two patients received EGFR tyrosine kinase inhibitors (EGFR-TKIs) during their treatment within our data collection period. Patient #1 was a non-smoking woman with adenocarcinoma. She received first-line cisplatin plus pemetrexed and had disease progression six months later. Her NGS test revealed an EGFR exon 20 insertion A763_Y764insFQEA, and she received erlotinib as second-line therapy. Her best response to EGFR-TKI was stable disease, and she remained on treatment for 5.9 months. The OS was 19.9 months. Patient #7 was another non-smoking woman with adenocarcinoma. The first-line treatment was cisplatin plus pemetrexed. She received erlotinib as a second-line treatment, and the best response was stable disease. The treatment continued for 10.1 months. Later, she underwent serial salvage chemotherapy regimens, including vinorelbine and carboplatin plus paclitaxel. Due to disease progression to multiple lines of treatment, the NGS test was arranged using the specimen from her initial diagnosis. It revealed an uncommon EGFR exon 19 deletion T751_I759 > S. Since she had been exposed to first-generation EGFR-TKIs, we arranged a rebiopsy for cobas EGFR mutation test. The result showed negative for her origin uncommon exon 19 deletion though, but founded an acquired resistance T790M mutation. She received osimertinib as the fifth-line treatment, with the best response of stable disease, which was maintained for 10.9 months. Her OS was 33.8 months. The other patient with exon 19 deletion (Patient#5) was still under her first-line chemotherapy at that moment. The above information is summarized in Table 2.

## Discussion

Using NGS, we found that the cobas EGFR test had a false-negative rate of up to 11.3%. In our previous study, we reported a false-negative rate of 8.1% for the same test using Sanger sequencing^18^. This study confirmed that the false-negative rate of a test would be higher when using a more sensitive method as a standard. In this cobas EGFR-negative cohort, most missed EGFR mutations were exon 20 insertions, followed by uncommon exon 19 deletions. Most of these mutations were not included in the coverage area of the pre-designed primers in the cobas system^16^. Patients with these missed uncommon EGFR mutations may still benefit from EGFR inhibitors with a durable response, especially the patients with exon 20 insertions since several effective therapies for them are available. Additionally, many patients were detected with actionable driver mutations, such as ROS1, BRAF, MET exon 14 skipping, and KRAS G12C using NGS. A more comprehensive gene study could identify more targetable candidates. To our knowledge, this is the first real-world report of the intrinsic limitations of the cobas EGFR test using NGS in areas with a high prevalence of EGFR mutations.

The clinical performance of the cobas test and NGS has been evaluated previously^21–24^. Both the positive and negative agreement rates were over 90% in detecting exon 19 deletions and L858R. A total of 408 samples from the EURTAC trial were included in the agreement analysis. The false-negative rate of the cobas EGFR test was 1.3% (4/313) for exon 19 deletion, 1.4% (5/355) for L858R, and 3.46% (9/260) for both exon 19 deletion and L858R^25^. Our study presented a similar false-negative rate for exon 19 deletion and L858R combined (2/62, 3.2%). Most other missed EGFR mutations were exon 20 insertions, which is an intrinsic limitation of the cobas test. In a cohort with a high prevalence of EGFR mutations, these intrinsic limitations accounted for a high proportion of false negatives. This information is important for physicians interpreting patients’ NGS results.

Exon 20 insertions account for about 4%–10% of all mutations in EGFR-mutant NSCLC, varying between different study populations and mutation detection platforms^26^. Reportedly, the frequency of exon 20 insertions in EGFR-mutant NSCLC was approximately 7.6% using NGS on US-based genomic databases^26,27^. In Taiwan, exon 20 insertions accounted for 4.0% of all EGFR-mutant NSCLC cases using direct sequencing^28^. The frequency of exon 20 insertions may have been underestimated because of detection limitations^26^. Baumi et al. had shown that PCR tests could miss about 50% exon 20 insertions due to the limitation of pre-designed commercial PCR kits compared with NGS study^27^. In our study, exon 20 insertions represented 8.1% (5/62) of the total mutations in the cobas EGFR-negative cohort. Exon 20 insertions are known to have worse outcomes. NSCLC harboring exon 20 insertions showed poor response to traditional EGFR-TKIs, and most patients received chemotherapy with suboptimal survival. However, exon 20 insertion variants were heterogeneous in both molecular and clinical presentations^27,29^. For example, A763_Y764insFQEA showed a favorable response to EGFR-TKIs^28,30,31^. One of our patients was A763_Y764insFQEA and did benefited from EGFR-TKIs with durable response. As more NGS studies are conducted, more details about exon 20 insertions will be discovered. Besides, several clinical trials have made significant breakthroughs in exon 20 insertions. Novel targeted therapies, such as amivantamab, mobocertinib, CLN-081, BDTX-189, and DZD9008, have shown response rates between 35 and 40%. FDA has recently approved amivantamab and mobocertinib for clinical use^26^. Amivantamab is a EGFR-MET bispecific antibody and promotes cellular cytotoxicity by natural killer cells. It has been reported with an overall response rate 40% and a median duration of response of 11.1 months in patients with exon 20 insertion and progression after platinum chemotherapy^32^. Mobocertinib is a TKI targeting exon 20 insertions. It has been confirmed with objective response rate of 28% and median progression-free survival of 7.3 months in phase 1/2 trial^33^. Comparing to conventional second-line docetaxel, which showed response rate 14%, both two drugs reported promising outcome. The FDA also approved NGS-based companion diagnostic for exon 20 insertion^34,35^. Precise sequencing methods and treatment strategies can meet the need for efficacious treatment for exon 20 insertions.

The false-negative rate of EGFR mutation (11.3%) was even higher than some driver mutations; and therefore deserves more attention. Approximately 40% of NSCLC patients in this EGFR/ALK double wild-type cohort had driver mutations with FDA-approved treatments. This indicates that a high proportion of NSCLC patients may benefit from a comprehensive molecular analysis, and every actionable driver mutation should be discovered. Current clinical practice requires several singleplex tests, including allele-specific real-time PCR assays for EGFR detection and immunohistochemical staining or fluorescence in situ hybridization for ALK and ROS1 rearrangement. However, this stepwise approach requires a higher quantity of tissue and a longer turnaround time^20^. Despite its shorter turnaround time and greater mutation coverage, the higher cost and demand for tissue samples are the main limitations of NGS testing^36–38^. Nevertheless, the varied prevalence of EGFR mutations in different areas plays an important role in decision-making. Further cost-effectiveness evaluations are required. Currently, whether to choose NGS study or standard single-gene molecular testing still rely on the pre-analytical management of specimens and the impact of the therapeutic plan^39^.

Our study has several limitations. First, this was a retrospective cohort study with its inherent limitations, especially the accuracy of the data on clinical conditions. Some laboratory parameters, such as DNA qualification and quantification data, may not be obtained due to our retrospective setting. Second, in this study, the allele-specific real-time PCR assay and NGS test might not have used the same clinical samples as in our previous study^18^. This was mainly due to the high tissue requirement for the NGS test. Although about 77.4% of specimens were from the same clinical samples for both cobas EGFR test and NGS test, there is still concern of treatment-induced modification of tumor characteristics. Third, we focus on the intrinsic limitation of lacking pre-designed primers for rare mutations in cobas test. However, the mutant allele frequency may also affect the concordance rate. Different mutations, such as base substitutions and indels, showed different limitation of detection. Lastly, NGS requires higher tumor content and protocolized DNA quantification. The high failure rate (28.7%) due to inadequate DNA extraction may also mislead the false-negative rate. However, this study has its strengths. First, all patients underwent the same NGS test with pre-testing quality control. The protocol was not only standardized but also approved for clinical practice by the US FDA. Likewise, all patients were defined as “wild-type EGFR” using the same allele-specific real-time PCR assay. This control ensured a better comparison between the two tests. Furthermore, all samples for the genetic study were FFPE pathology samples, and no liquid biopsy was included, which showed differences in sensitivity in detecting mutations. Therefore, our results represent real-world evidence and can be directly applied to clinical settings.

## Conclusions

Using NGS as the standard, the false-negative rate of the EGFR mutation test by allele-specific real-time PCR was 11.3%. The most overlooked mutations were exon 20 insertions. These patients may still benefit from EGFR inhibitors. A sensitive and comprehensive EGFR mutation assay can provide valuable information to NSCLC patients, especially in areas with a high prevalence of EGFR mutations.

## Data Availability

The datasets generated during and/or analyzed during the current study are not publicly available due to patients’ privacy but are available from the corresponding author on reasonable request.
